# Genetic causes of nephrolithiasis and nephrocalcinosis in a pediatric population in Saudi Arabia

**DOI:** 10.1007/s00467-025-07018-3

**Published:** 2025-11-07

**Authors:** Hadel Alsubaie, Bashaer Alluhaybi, Faten Zaidan, Zuhair Rahbeeni, Essam Alsabban, Turki Alshareef, Weiam Almaiman, Raghad Alhuthil, Sermin Saadeh

**Affiliations:** 1 Pediatric Nephrology, Department of Pediatrics, King Faisal Specialist Hospital and Research Centre, Riyadh, Saudi Arabia; 2 Medical Genomics Department, King Faisal Specialist Hospital and Research Centre, Riyadh, Saudi Arabia; 3 Clinical Sciences Department, College of Medicine, Almaarefa University, Riyadh, Saudi Arabia

**Keywords:** Nephrocalcinosis, Nephrolithiasis, Mongenic, *CLD16*

## Abstract

**Background:**

Nephrolithiasis (NL) and nephrocalcinosis (NC) are common, recurrent conditions globally. While monogenic causes are increasingly recognized, data on their prevalence and spectrum remain limited in Saudi pediatric populations.

**Methods:**

This retrospective cross-sectional study was conducted in our tertiary care center from January 2008 to April 2023. Pediatric patients (0–18 years) with radiologically confirmed NL/NC who underwent genetic testing were included. Clinical, biochemical, radiological, and genetic data were analyzed. Genetic variants were classified using ACMG criteria, and segregation analysis was performed when available.

**Results:**

Of 186 pediatric patients diagnosed with NL/NC, 54 (29.03%) underwent genetic testing. Median age at diagnosis was 3 months [IQR: 3–60], with median follow-up 56 months [IQR: 24–108]. Genetic mutations related to NL/NC were identified in 35/54 patients (64.81%), most commonly in *CLDN16* (28.57%), *SLC2A2* (17.14%), *AGXT* (11.43%), and *SLC12A1* (8.57%). Thirteen novel variants were identified, with eleven linked to NL/NC phenotypes. Eight patients (14.81%) developed kidney failure requiring kidney replacement therapy; *CLDN16* was significantly associated with kidney failure and transplant (*P* = 0.003), and *AGXT* with liver transplant (*P* < 0.001). Notably, the *MOCS1* gene was found in a patient with early-onset neurological symptoms, hypouricemia, and later confirmed NL/NC.

**Conclusion:**

Monogenic causes were identified in 35 of 54 (64.81%) Saudi pediatric patients with NL/NC who underwent genetic testing, a prevalence higher than reported internationally, likely due to the high consanguinity rate. Our findings underscore the importance of genetic testing in early-onset NL/NC. We recommend adding *MOCS1* to the list of genes associated with monogenic NL/NC.

**Graphical abstract:**

**Figure:**
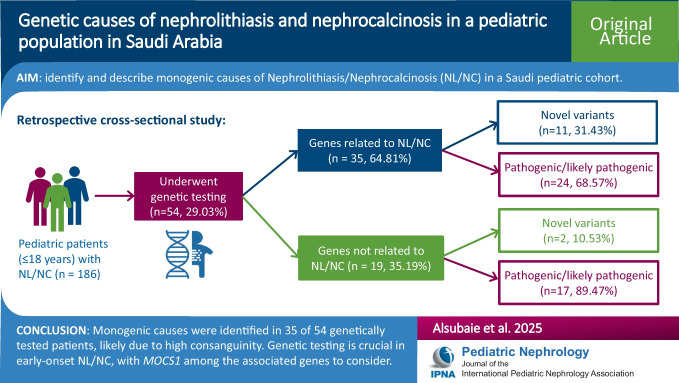
A higher resolution version of the Graphical abstract is available as Supplementary information

**Supplementary Information:**

The online version contains supplementary material available at 10.1007/s00467-025-07018-3.

## Introduction

Nephrolithiasis (NL) and nephrocalcinosis (NC) are common pediatric kidney conditions characterized by high recurrence rates and increasing global prevalence [1–3]. In Saudi Arabia, the local prevalence of NL/NC has been reported to range between 9.1% and 33.1% [4, 5]. While global trends indicate a rising incidence of kidney stones in children—rising from 6 to 10% over the past two decades—local data on pediatric NL/NC remain limited [6, 7]. Furthermore, diagnostic approaches vary considerably across institutions, affecting the comparability of incidence estimates.

Although glomerular diseases are the most common cause of pediatric kidney failure, hereditary and congenital disorders account for approximately 12.7% of cases in children, according to the United States Renal Data System [8, 9]. In children with NL/NC, the prevalence of monogenic causes has been estimated at approximately 30% [10]. However, estimations vary widely depending on selection criteria, diagnostic techniques, and population genetics. Genetic evaluation is increasingly recognized as a critical tool in cases lacking a clear family history or presenting with early or recurrent disease. For instance, Benito et al. reported a case of autosomal dominant distal renal tubular acidosis diagnosed only after genetic testing in a young girl with NC and no family history of kidney disease [2].

Several studies underscore the value of early genetic testing in identifying causative mutations, enabling earlier intervention, screening of at-risk relatives, and discovery of novel disease-associated genes [2, 11–15]. Populations with high consanguinity, such as in Saudi Arabia, present a unique opportunity to explore monogenic kidney disease — particularly recessive forms — due to the increased likelihood of homozygous pathogenic variants [15, 16].

To facilitate clinical diagnosis, commercial genetic testing panels for monogenic NL/NC now include over 30 known genes [12, 17, 18]. These include *ADCY10*, *AGXT*, *APRT*, *ATP6V0A4*, *ATP6V1B1*, *CA2*, *CASR*, *CLCN5*, *CLCNKB*, *CLDN16*, *CLDN19*, *CYP24A1*, *FAM20A*, *GRHPR*, *HNF4A*, *HOGA1*, *HPRT1*, *KCNJ1*, *OCRL*, *SLC12A1*, *SLC22A12*, *SLC2A9*, *SLC34A1*, *SLC34A3*, *SLC3A1*, *SLC4A1, SLC7A9*, *SLC9A3R1*, *VDR*, and *XDH* [12]. Additional genes, such as *SLC26A1*, *PRPS1*, and *MOCS1*, have also been implicated but are not routinely included in standard panels [19, 20]. To improve diagnostic yield, consideration of these emerging genes in targeted panels may be warranted, especially in high-risk populations.

Despite growing awareness of monogenic NL/NC, studies examining the prevalence and spectrum of these conditions remain scarce. This study aims to identify and describe monogenic causes of NL/NC in a Saudi pediatric cohort seen at a tertiary care center, with a focus on genetic patterns, novel variants, and genotype–phenotype correlations.

## Methods

This retrospective, cross-sectional study was conducted at King Faisal Specialist Hospital and Research Centre (KFSHRC), Riyadh, Saudi Arabia. Medical records from January 2008 to April 2023 were reviewed to identify pediatric patients (aged 0–18 years) with radiologically confirmed NL and/or NC.

Patients were included if they had a primary diagnosis of NL or NC based on ultrasound or other imaging studies and had undergone genetic testing as part of routine clinical evaluation. Patients with secondary causes of NL/NC, such as hyperparathyroidism, prematurity-related NC, or medication-induced stones, were excluded.

Data were retrieved from the hospital’s electronic medical records system and included demographics, clinical characteristics, biochemical findings, genetic testing results, radiological findings, transplant history, and mortality status. Genetic testing was performed at accredited local and international laboratories and included whole exome sequencing (WES), targeted gene panels, or whole genome sequencing, based on clinical indication and availability. The selection of test type was dependent on the availability and clinical suspicion, with WES favored in undiagnosed multisystemic cases.

The diagnosis of monogenic NL/NC was based on a combination of clinical features, radiological confirmation, biochemical abnormalities, and genetic evidence. Variants of uncertain significance (VUS) were assessed using family segregation studies, ClinVar [21], OMIM database [22], and genotype–phenotype correlation. Variants were categorized according to the American College of Medical Genetics and Genomics (ACMG) classification [23].

Statistical analyses were conducted using STATA version 17. Continuous variables were presented as medians with interquartile ranges (IQRs), and categorical variables as frequencies and percentages. The Wilcoxon signed-rank test was used to compare estimated glomerular filtration rate (eGFR) at diagnosis and at last follow-up. Fisher’s exact test was used to assess associations between gene mutations and clinical outcomes. A *P*-value < 0.05 was considered statistically significant. No adjustments for multiple comparisons were applied.

The study was approved by the Institutional Review Board of KFSHRC (reference number: 2201105). Genetic testing was conducted as part of routine clinical care.

## Results

**Fig. 1:**
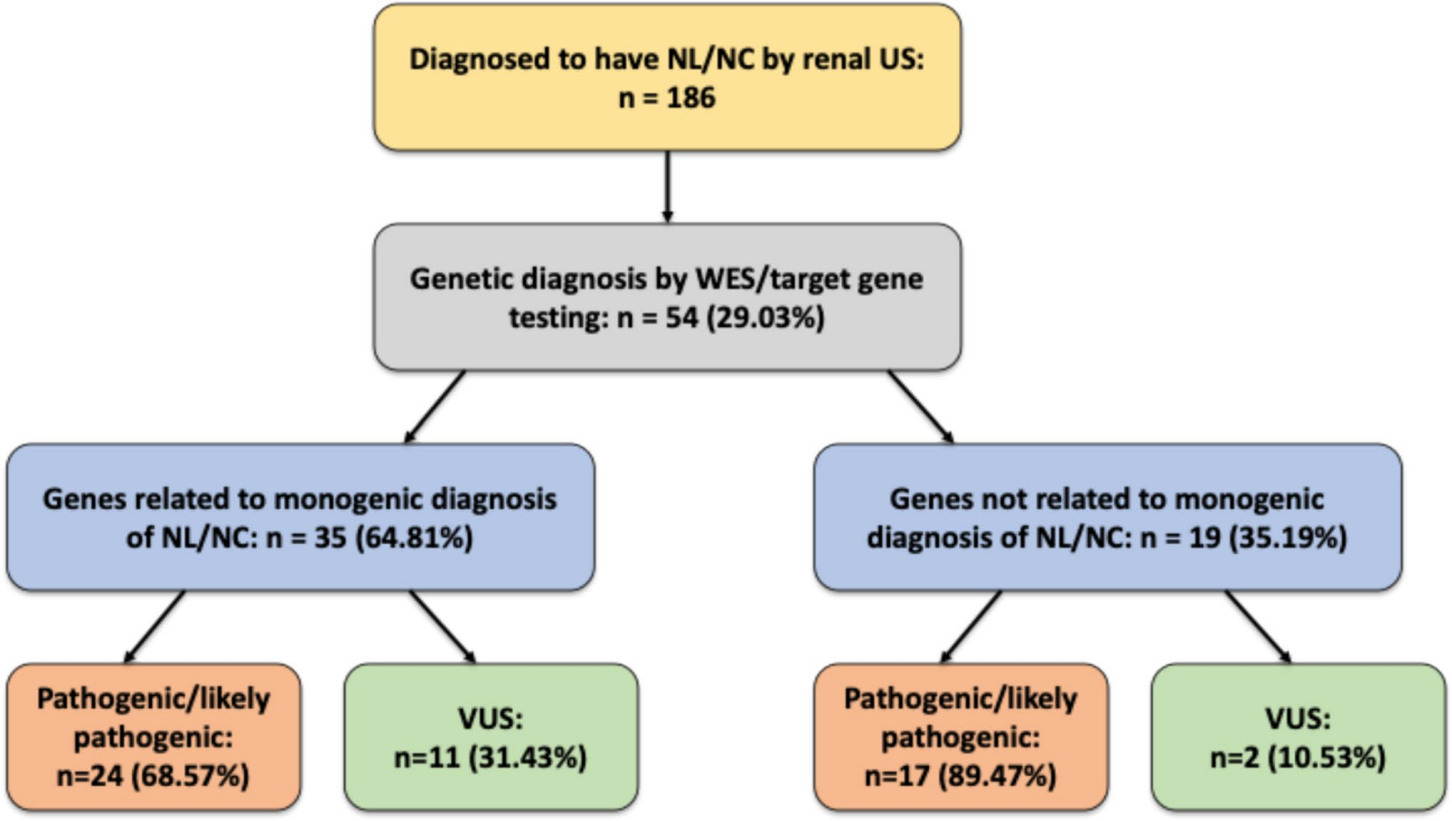
Flow diagram of the pediatric Nephrolithiasis/Nephrocalcinosis (NL/NC) study cohort. US: ultrasound, WES: whole exome sequencing, VUS: variant of uncertain significance

Of the 186 pediatric patients diagnosed with NL or NC at our center during the study period, 54 (29.03%) underwent genetic testing and were included in the analysis (Fig. 1). Among these, 34 (62.96%) were male, with a median age at diagnosis of 3 months [IQR: 3–60], and the median follow-up duration was 56 months [IQR: 24–108]. Table 1 summarizes the baseline characteristics of the cohort.

**Table 1:** Baseline characteristics of patients (*n* = 54)

Characteristics	*n* (%), Median [IQR]
Sex	
Male	34 (62.96)
Female	20 (37.04)
Age at diagnosis (months)	3 [3–60]
Age at last follow up (months)	48 [48–156]
Duration of follow-up (months)	56 [24–108]
Renal ultrasound findings	
Nephrocalcinosis	40 (74.07)
Nephrolithiasis	6 (11.11)
Both	8 (14.81)
Genetic test	
Whole Exome Sequencing	19 (35.19)
Target Gene Mutation	28 (51.85)
Whole-genomic sequencing	1 (1.85)
Unknown	6 (11.11)
Kidney Failure/Kidney Replacement Therapy	8 (14.81)
Age at KRT (years)	12.1 [8.5–15.4]
Kidney transplant	5 (9.26)
Liver transplant	5 (9.26)
Mortality	5 (9.26)

Monogenic causes of NL/NC were identified in 35 patients (64.81%) (Fig. 1). The most commonly mutated genes were: *CLDN16* (10 patients, 28.57%), *SLC2A2* (6 patients, 17.14%), *AGXT* (4 patients, 11.43%), and *SLC12A1* (3 patients, 8.57%). Other implicated genes included *ATP6V0A4*, *CLDN19*, *CYP24A1*, *CaSR*, *HPRT1*, *KCNJ1*, *SLC26A1*, *CTNS*, *MOCS1*, *SLC34A1*, *ALPL*, and *CDC73* (each found in one patient, 2.86%). Homozygous mutations accounted for 91.43% of cases with identified monogenic causes (Table 2).

**Table 2:** Causative genetic mutations (*n* = 35)

Gene mutation	Disease phenotype	Zygosity status	*n* (%)
*AGXT*	Primary Hyperoxaluria Type 1 (PH1)	Homozygous in 3 Compound heterozygous in 1	4 (11.43)
*ATP6V0A4*	Renal Tubular Acidosis Type 4	Homozygous	1 (2.86)
*ALPL*	Hypophosphatasia	Homozygous	1 (2.86)
*CDC73*	Hyperparathyroidism-jaw tumor syndrome	Heterozygous	1 (2.86)
*CLDN16*	Familial Hypomagnesemia with Hypercalciuria and Nephrocalcinosis (FHHNC)	Homozygous	10 (28.57)
*CLDN19*	Familial Hypomagnesemia with Hypercalciuria and Nephrocalcinosis (FHHNC)	Homozygous	1 (2.86)
*CYP24A1*	Idiopathic Infantile Hypercalcemia Type 1	Homozygous	1 (2.86)
*CaSR*	Familial Hypocalciuric Hypercalcemia Type 1 (FHH1)	Homozygous	1 (2.86)
*HPRT1*	Lesch-Nyhan Syndrome (LNS)	Homozygous	1 (2.86)
*KCNJ1*	Bartter Syndrome Type II	Homozygous	1 (2.86)
*MOCS1*	Molybdenum Cofactor Deficiency Type A	Homozygous	1 (2.86)
*SLC12A1*	Bartter Syndrome Type I	Homozygous	3 (8.57)
*SLC34A1*	Range of clinical phenotypes including infantile hypercalcemia, a proximal renal Fanconi syndrome, which are typically autosomal recessive, and hypophosphatemic nephrolithiasis	Homozygous	1 (2.86)
*SLC2A2*	Fanconi-Bickel syndrome	Homozygous	6 (17.14)
*CTNS*	Cystinosis	Homozygous	1 (2.86)
*SLC26A1*	Congenital anion exchanger deficiency	Heterozygous	1 (2.86)

Conversely, 19 patients (35.19%) had no identifiable monogenic cause of NL/NC despite undergoing genetic testing. Their detected variants were considered unrelated to NL/NC based on clinical phenotype and available evidence (Table 3). These included mutations in genes commonly linked to syndromic or unrelated metabolic disorders.

**Table 3:** Non-causative genetic mutation (*n* = 19)

Gene mutation	Phenotype/disease	Nephrocalcinosis present (y/n)	*n* (%)
*SLC26A3*	Congenital chloride diarrhea	Y	2 (10.53)
*GSD1A*	Glycogen Storage Disease Type 1A	N	1 (5.26)
*ABCB4*	Progressive familial intrahepatic cholestasis type 3	N	1 (5.26)
*AIRE*	Autoimmune polyendocrine syndrome type 1	N	1 (5.26)
*AME*	Amelogenesis imperfecta	N	1 (5.26)
*ARID1B*	Coffin-Siris syndrome	N	1 (5.26)
*CFHR5*	C3 glomerulopathy	N	1 (5.26)
*CYP21A2*	Congenital adrenal hyperplasia	Y	1 (5.26)
*CYP27B1*	Vitamin D-dependent rickets type 1	Y	1 (5.26)
*CYP7B1*	Smith-Lemli-Opitz syndrome	N	1 (5.26)
*DCLRE1C*	Severe combined immunodeficiency (SCID)	N	1 (5.26)
*KCNJ11*	Neonatal diabetes mellitus	N	1 (5.26)
*MRPL44*	Mitochondrial dysfunction	N	1 (5.26)
*MTM1*	X-linked myotubular myopathy	N	1 (5.26)
*PHEX*	X-linked hypophosphatemia	Y	1 (5.26)
*POLG*	Mitochondrial DNA depletion syndrome	N	1 (5.26)
*PTEN*	Cowden syndrome	N	1 (5.26)
*STXBP2*	Familial hemophagocytic lymphohistiocytosis type 5	N	1 (5.26)

*Y*, Yes, *N*, No

Metabolic abnormalities were observed in 42 out of 54 patients (77.78%). The most common abnormality was hypercalciuria (36 patients, 85.71%), followed by hyperoxaluria, hypophosphatemia, hypomagnesemia, and other disturbances (Table 4). In some patients, multiple metabolic abnormalities were noted concurrently.

**Table 4:** Metabolic abnormalities (*n* = 42)

Metabolic abnormalities	*n* (%)
Hypercalciuria	36 (85.71)
Hyperoxaluria	3 (7.14)
Hypophosphatemia	3 (7.14)
Metabolic alkalosis	3 (7.14)
Hypomagnesemia	3 (7.14)
Hypokalemia	2 (4.76)
Hyponatremia	2 (4.76)
Hyperparathyroidism	2 (4.76)
Hypercalcemia	2 (4.76)
Glucosuria	1 (2.38)
Proteinuria	1 (2.38)

Eight patients (14.81%) progressed to kidney failure requiring kidney replacement therapy (KF/KRT); these included cases with homozygosity in *CLDN16* (*n* = 5), *AGXT* (*n* = 2), and *CLDN19* (*n* = 1). Of these eight, five underwent kidney transplantation (see Supplementary File 1).

Statistical analysis revealed significant associations between specific gene mutations and adverse outcomes. *CLDN16* mutations were significantly associated with both kidney failure/kidney replacement therapy (KF/KRT) and kidney transplantation (*P* = 0.003 for both). *AGXT* mutations were significantly associated with liver transplantation (*P* < 0.001) (Table 5).

**Table 5:** Genes associated with poor outcomes

**Gene**	**Liver transplant**	**No liver transplant**	**Total**	***P*-value**
***AGXT***	4/5 (80.00)	0/49 (0.00)	4/54 (7.41)	< 0.001*
	**Kidney transplant**	**No kidney transplant**	**Total**	***P*-value**
***CLDN16***	4/5 (80.00)	6/49 (12.24)	10/54 (18.52)	0.003*
	**KF/KRT**	**Non-KF/KRT**	**Total**	***P*-value**
***CLDN16***	5/8 (62.50)	5/46 (10.87)	10/54 (18.52)	0.003*

Data are presented as *n* (%). *P*-values were determined using Fisher’s exact test

*KF/KRT*, Kidney Failure/Kidney Replacement Therapy

^*^Indicates statistical significance < 0.05

Thirteen patients (24.07%) were found to have novel genetic variants, of which eleven were associated with NL/NC. A recurrent novel mutation in *CLDN16* (c.362 T > G, p.Val121Gly) was identified in four patients — three siblings and one unrelated patient from the same tribe. Additional novel variants were found in *AGXT*, *SLC12A1*, *KCNJ1*, *CaSR* and *SLC26A1*, as detailed in Table 6. The overall mortality rate was 9.3% (5/54) (Table 1).

**Table 6:** Novel variants found in 13 patients

#	Molecular test	Type of variant	*n*	Functional test/segregation	ClinVar	Pathogenicity summary
***Non-causative genes***						
1	*KCNJ11*	c.472A > T (homozygous)	1	Parents heterozygous	Not reported	Genotype–phenotype correlation: not associated with nephrocalcinosis/nephrolithiasis in OMIM
2	*ARID1B*	c.4855delG (heterozygous)	1	De novo	Not reported	Genotype–phenotype correlation: not associated with nephrocalcinosis/nephrolithiasis in OMIM
***Causative genes***						
1	*SLC12A1*	c.1834G > A (homozygous)	1	Parents heterozygous	Likely pathogenic	Confirmed by phenotype–genotype correlation and ACMG criteria
2	*KCNJ1*	c.297G > T and c.299A > T (compound heterozygous)	1	Parents were heterozygous for two mutations	Pathogenic for c.297G > T	Phenotype–genotype correlation, segregation and OMIM documentation
3	*SLC12A1*	c.1166C > T (homozygous)	1	Parents and sibling are heterozygous	Not reported	Phenotype–genotype correlation and OMIM documented
4	*AGXT*	c33dupC c584T > G c.590G > A (compound heterozygous)	1	Parents were heterozygous for two mutations and her affected sibling homozygous for c.584T > G	Reported as pathogenic for c.584T > G	Segregation and OMIM documentation
5	*CLDN16*	c.362T > G (homozygous)	4	Three affected siblings homozygous for c.362T > G, and one unrelated patient from the same tribe	Not reported	Segregation and OMIM documentation
6	*CaSR*	c.2084G > T (homozygous)	1	Not done	Not reported	OMIM documented nephrocalcinosis/nephrolithiasis
7	*SLC26A1*	c.1772A > G (heterozygous)	1	Not done	Not reported	OMIM documents disease only in homozygous state
8	*SLC2A2*	c.117A > C (homozygous)	1	Not done	Not reported	Not documented in OMIM for nephrocalcinosis/nephrolithiasis

## Discussion

NL and NC in pediatric populations present diagnostic and therapeutic challenges due to their early onset, frequent recurrence, and diverse etiologies. Identifying the primary cause—particularly genetic forms—is critical for timely intervention, genetic counseling, and treatment planning. In this study, we investigated monogenic causes of NL/NC in a Saudi pediatric cohort, finding a relatively high prevalence of genetic diagnoses (64.81%), with notable contributions from *CLDN16*, *SLC2A2*, *AGXT*, and *SLC12A1*.

Our observed genetic prevalence exceeds that reported by Braun et al. (16.8%) [24], aligns closely with findings by Wang et al. (46.3%) [25], and is higher than the 29.4% reported by Daga et al. [26]. These differences likely reflect varying inclusion criteria, with both Wang et al. and Daga et al. selecting patients based on family history and excluding secondary causes, potentially inflating monogenic case proportions. Our higher diagnostic yield may also be attributed to the high consanguinity rates in the Saudi population, which increase the likelihood of autosomal recessive homozygous mutations [16, 27].

Sex distribution in our cohort was balanced among genetically affected individuals, in agreement with prior findings by Wang et al., Braun et al., and Daga et al. [24–26]. However, this contrasts with Alhasan et al. [27], who observed a higher male-to-female ratio for NL and a female predominance in NC. One-third of our patients were diagnosed before the age of one year, consistent with the typical earlier onset of autosomal recessive diseases.

In our cohort, *CLDN16* was the most frequently mutated gene, representing 31.25% of genetically linked cases. The recurrent novel mutation c.362 T > G (p.Val121Gly) was found in four patients, three of whom were siblings, and the fourth from the same tribe. All ten patients with *CLDN16* mutations were homozygous. This finding contrasts with other studies: Wang et al. reported *SLC3A1* as the most common mutation [25], Halbritter et al. identified *SLC7A9* [18], and Daga et al. found *SLC34A1* to be most prevalent [26]. None of the mutations identified in our study were autosomal dominant, which is consistent with the pattern observed in consanguineous populations.

Our findings also reinforce the clinical significance of *AGXT* mutations, particularly their strong association with liver transplantation (*P* < 0.001). Additionally, *CLDN16* mutations were significantly associated with both KF/KRT and kidney transplantation (*P* = 0.003). These results support the utility of gene-specific risk profiling in disease progression. Risk stratification models incorporating genotype may help guide early referral and organ-specific surveillance.

A significant contribution of this study is the identification of 13 novel genetic variants, eleven of which were linked to the NL/NC phenotype. Pathogenicity was supported through phenotype–genotype correlation, family segregation analysis, and ACMG classification. Importantly, the *MOCS1* gene was found in a patient with early-onset neurological symptoms, hypouricemia, and later confirmed NL/NC. Although *MOCS1* is typically associated with molybdenum cofactor deficiency (MoCoD) and xanthinuria, it is rarely considered in the context of NL/NC. Prior reports by Bayram et al. and Lee et al. have described similar presentations [28, 29], but this is one of the first studies to suggest *MOCS1* as a gene that may directly contribute to NL/NC pathogenesis. Its inclusion in future diagnostic panels may enhance early identification and management of this rare condition.

One of our patients was found to have a newly identified gene, *SLC26A1*, linked to NL/NC. We detected a heterozygous missense mutation (c.1772A > G, p.Glu591Gly) in this patient, who presented at 5 years of age with high urinary calcium oxalate levels and persistent mild medullary NC. Despite these findings, the patient has maintained normal kidney function throughout the follow-up period. The heterozygous nature of the mutation may explain the relatively mild disease course.

This case contrasts with the report by Gee et al., who described two unrelated individuals with calcium oxalate kidney stones. One of these patients developed acute kidney failure secondary to recurrent obstructed kidney calculi. Genetic analysis of this patient revealed a compound heterozygous mutation in *SLC26A1* (c.554C > T, p.Thr185Met and c.1073C > T, p.Ser358Leu). The second patient, however, had normal kidney function. We hypothesize that the severity of the clinical presentation may be influenced by the nature of the gene mutation, with our patient having a heterozygous mutation that potentially results in a milder clinical course [19].

Although a few genes are not currently classified as causative for NL and NC, they are frequently reported in the literature as being associated with these conditions. These genes may exert indirect effects through metabolic dysregulation, tubular dysfunction, or mineral transport anomalies. Notable genes include *SLC26A3*, *CYP21A2*, and *CYP27B1* [30–34].

Clinicians should be mindful of the possibility of NL/NC in these patients, regularly monitoring kidney function and conducting appropriate imaging when indicated. Early recognition and ongoing surveillance of NL/NC can help mitigate complications such as kidney damage, kidney stones, and chronic kidney disease, ultimately improving patient outcomes. By integrating these genes into databases and clinical guidelines, we can ensure that practitioners are equipped to identify and manage NL/NC more effectively, enhancing care for affected individuals.

This study is limited by its retrospective, single-center design and the absence of a control group, which restricts the ability to determine the relative contribution of genetic factors compared with other etiologies. Additionally, data on dietary, environmental, and 24-h urine metabolic assessments were inconsistently available and therefore not analyzed, limiting insights into gene–environment interactions in NL/NC pathogenesis. Functional validation of several variants of uncertain significance was not possible due to resource constraints. Nonetheless, the study's strengths include its relatively large cohort size, long-term follow-up, and focus on a genetically unique population.

## Conclusion

This study identified monogenic causes of NL and NC in 35 of 54 (64.81%) of Saudi pediatric patients who underwent genetic testing, a rate higher than typically reported internationally. The most common genetic contributors were *CLDN16*, *SLC2A2*, *AGXT*, and *SLC12A1*, with a predominance of homozygous variants reflecting the high consanguinity in the region. Notably, while *MOCS1* is primarily associated with MoCoD and xanthinuria, it has rarely been implicated in the context of NL/NC. These findings highlight the importance of genetic testing in early-onset or unexplained pediatric NL/NC and support the inclusion of *MOCS1* in monogenic NL/NC gene panels. Further multicenter studies and functional analyses, including in vitro studies and animal models, are needed to expand our understanding of genotype–phenotype correlations in this population.

## Supplementary Information

Below is the link to the electronic supplementary material.

ESM 1 Graphical abstract (PPTX 91.4 KB)

ESM 2 (DOCX 28.4 KB)

## Data Availability

All data generated or analyzed during this study are included in this published article. Additional data are available from the corresponding author upon reasonable request.
