# Supplementary material for: Genetic causes of nephrolithiasis and nephrocalcinosis in a pediatric population in Saudi Arabia
Source: Pediatr Nephrol. 2025 Nov 7;41(3):691–8. doi: 10.1007/s00467-025-07018-3 (PMC12852217; doi:10.1007/s00467-025-07018-3)
Supplement: Supplementary file 1 — Graphical abstract (PPTX 91.4 KB) [file 467_2025_7018_MOESM1_ESM.pptx]

## Slide 1
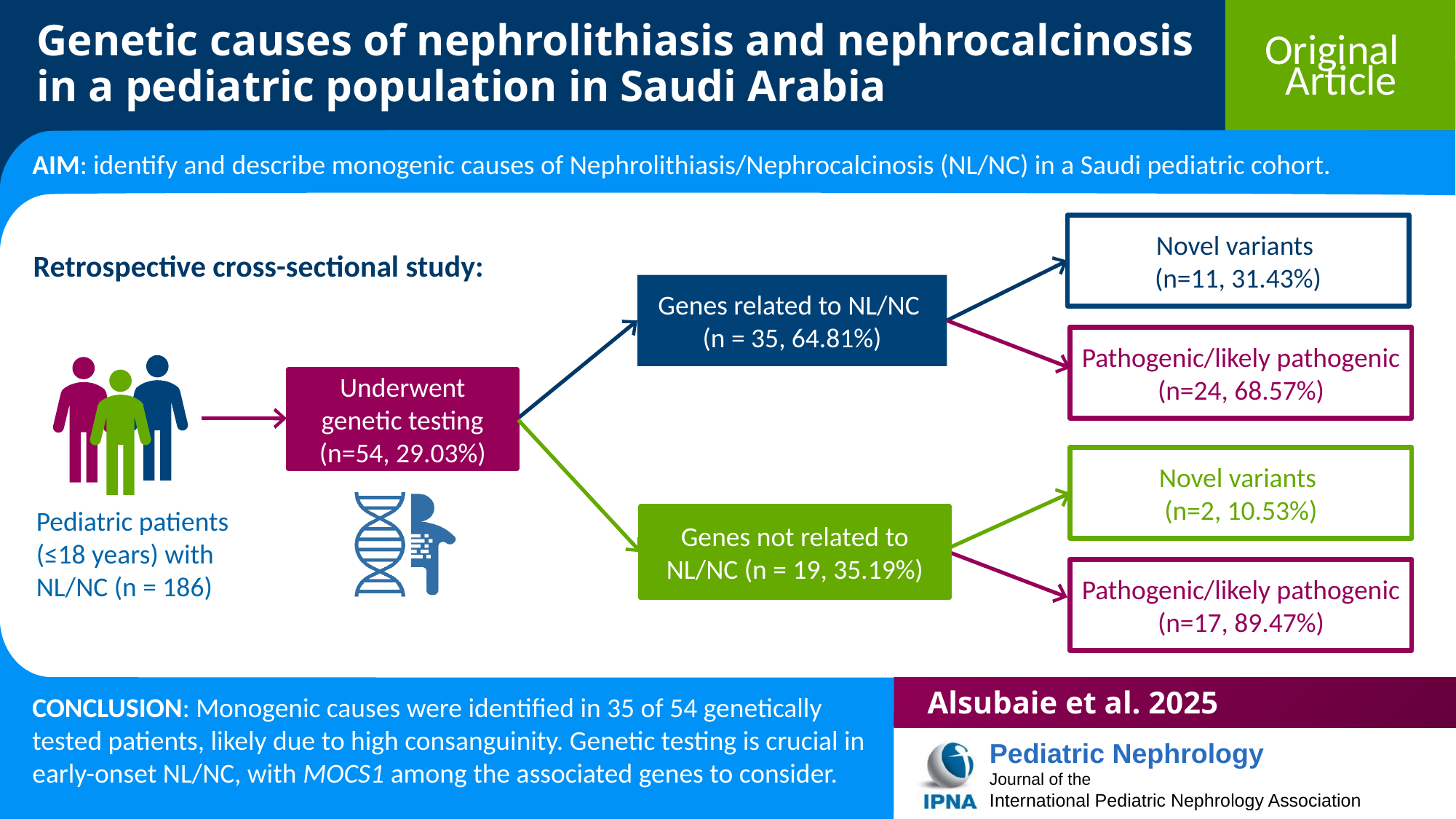

Genetic causes of nephrolithiasis and nephrocalcinosis in a pediatric population in Saudi Arabia
AIM: identify and describe monogenic causes of Nephrolithiasis/Nephrocalcinosis (NL/NC) in a Saudi pediatric cohort.
Retrospective cross-sectional study:
Novel variants
(n=11, 31.43%)
Genes related to NL/NC
(n = 35, 64.81%)
Pathogenic/likely pathogenic (n=24, 68.57%)
Underwent genetic testing (n=54, 29.03%)
Novel variants
(n=2, 10.53%)
Pediatric patients (≤18 years) with NL/NC (n = 186)
Genes not related to NL/NC (n = 19, 35.19%)
Pathogenic/likely pathogenic (n=17, 89.47%)
Alsubaie et al. 2025
CONCLUSION: Monogenic causes were identified in 35 of 54 genetically tested patients, likely due to high consanguinity. Genetic testing is crucial in early-onset NL/NC, with MOCS1 among the associated genes to consider.
