# Supplementary material for: Genetic causes of nephrolithiasis and nephrocalcinosis in a pediatric population in Saudi Arabia
Source: Pediatr Nephrol. 2025 Nov 7;41(3):691–8. doi: 10.1007/s00467-025-07018-3 (PMC12852217; doi:10.1007/s00467-025-07018-3)
Supplement: Supplementary file 2 — (DOCX 28.4 KB) [file 467_2025_7018_MOESM2_ESM.docx]

**Supplementary information**

**Supplementary Table 1. Clinical characteristics of patients with kidney failure (n = 8)**

| **Case No.** | **Gender** | **Gene mutation** | **Age at diagnosis (yr)** | **Age at dialysis (yr)** | **Type of transplant** | **Outcome** |
| --- | --- | --- | --- | --- | --- | --- |
| 1 | F | AGXT | 7.0 | 17.7 | Liver | Alive |
| 2 | F | AGXT | 1.5 | 5.5 | Liver | Died |
| 3 | M | CLDN16 | 3.0 | 11.2 | Kidney | Alive |
| 4 | M | CLDN16 | 6.0 | 10.0 | Kidney | Alive |
| 5 | M | CLDN16 | 9.0 | 13.1 | Kidney | Alive |
| 6 | M | CLDN16 | 11.0 | 12.9 | Kidney | Alive |
| 7 | F | CLDN16 | 12.0 | 18.0 | None | Alive |
| 8 | M | CLDN19 | 10.0 | 7.0 | Kidney | Alive |
